# Who elects the weekend?

**DOI:** 10.1371/journal.pmed.1002732

**Published:** 2019-01-29

**Authors:** Lauren Lapointe-Shaw, Chaim M. Bell

**Affiliations:** 1 Department of Medicine, University of Toronto, Toronto, Ontario, Canada; 2 Department of Medicine, Sinai Health System, Toronto, Ontario, Canada

## Abstract

Chaim M. Bell and Lauren Lapointe-Shaw discuss the meaning of the "weekend effect" in outcomes for hospital admissions and surgeries, and comment on surprising new results published in PLOS Medicine this week.

Who would have thought that outcomes for elective surgery would differ according to the day of admission? In this issue of *PLOS Medicine*, O’Leary and colleagues compare the outcomes of weekend-admitted patients undergoing surgery with those of weekday-admitted patients [1]. This study adds to a growing body of literature identifying worse outcomes in patients according to the timing of admission and/or surgery. O’Leary and colleagues studied the outcomes of over 1.3 million patient admissions to acute care hospitals in Ontario, Canada, from 2005 to 2015. Admissions without any surgical procedure, or with a cardiac, cardiothoracic, nonsurgical, or obstetric procedure were excluded. The results indicate that weekend-admitted surgical patients have a greater risk of 30-day mortality than surgical patients admitted on weekdays (2.6% versus 2.5%, adjusted OR 1.05; 95% CI, 1.00–1.11). The question arises whether the weekend effect reported by O’Leary and colleagues results from true differences in care or is reflective of major differences in baseline patient risk between weekend and weekday patients.

## Distinguishing patient and care effects

Since the weekend effect was first described, many have wondered whether it results from variation in care or simply differences in the risk profiles of weekend and weekday patients [2–4]. Methods to adequately account for possible confounders between weekend care and patient outcomes are essential to attribute differences in outcomes to differences in care. Yet, most studies of the weekend effect have relied on administrative data sources and thus have been limited to readily available measures of baseline risk, such as age, sex, comorbidity counts, and previous healthcare usage. These commonly included variables offer little information on severity of illness, as they are mostly limited to health status prior to the current presentation.

Because of the importance of ensuring equity of care across all days of the week, tools to untangle patient and care effects are in continuous development. For example, excess risk over time curves have been used to identify conditions with an early-peaking risk, which could signal an acute weekend care–related effect. In contrast, a long period of increased risk may be a marker of a higher risk patient profile [5]. Another approach is to include care process outcomes, as these offer a plausible mechanism whereby weekend care might lead to greater mortality [6–9]. In a further step, investigators can determine whether adding information on a process measure weakens the association between the timing of admission and a mortality outcome. As a recent example, lower rates of early invasive coronary angiography were found to explain the weekend effect observed in United States patients admitted to hospital for myocardial infarction [10]. Because the procedure occurs after the time of admission, it is plausible that the delay in procedure may mediate the weekend-outcome relationship. In contrast, if introducing additional baseline clinical information weakens the weekend-outcome association, then it is likely that the measured clinical characteristics are acting as confounders. The addition of more detailed clinical information gathered at the time of first presentation (for example, laboratory test results) has in many cases led to significant weakening, even disappearance, of the observed weekend effect [11–15]. This is because granular clinical information has often uncovered a greater severity of illness in weekend-admitted patients.

Differential admission patterns are thought to underlie the increased clinical severity of patients admitted to hospital on weekends [16]. A higher admission threshold on weekends leads to fewer but sicker weekend patients. One United Kingdom study found that fewer patients were admitted to hospital on weekends, despite stable emergency department volumes throughout the week [16]. The weekend-related drop in volume and the observed weekend effect on mortality are both more pronounced for elective, rather than urgent, admissions [17]. Overall, it is plausible that the further the weekend admission proportion deviates from 2/7, the greater the likelihood of major differences in weekend- and weekday-admitted patient characteristics, both measured and unmeasured.

## The weekend effect and surgical patients

Patients undergoing surgery on weekends have been found, in some cases, to experience worse outcomes than those operated on weekdays. Notably, studies of elective procedures have identified a significant weekend effect (with an odds ratio for mortality on the order of 1.8 or 1.9) [18,19], yet urgent procedures have demonstrated no such effect [20–22]. This finding is unexpected, as elective surgeries, by their planned nature, should be more likely to exhibit consistent outcomes throughout all days of the week. That the inverse occurs suggests a similar phenomenon to that observed for elective medical admissions. The large drop in elective surgical admissions and procedures on weekends may be introducing significant bias on the basis of differences in unmeasured severity of illness.

In the study reported in this issue, O’Leary and colleagues do an excellent job of examining the relationship between urgency of admission and the weekend effect for surgical patients. The association between weekend admission and slightly worse outcomes in the overall cohort can be explained by a large weekend effect for electively admitted patients. This may be greater for patients who undergo surgery on the weekend (adjusted OR 3.30; 95% CI, 1.98–5.49) than for those undergoing surgery on a subsequent weekday (adjusted OR 2.70; 95% CI, 1.81–4.03). In contrast, there was no weekend effect for urgently admitted patients, whether operated on the weekend (adjusted OR 1.02; 95% CI, 0.95–1.09) or on a subsequent weekday (adjusted OR 1.05; 95% CI, 0.98–1.12).

That electively admitted weekend patients were at risk regardless of the timing of surgery is noteworthy. For those undergoing surgery on a weekday, the “weekend effect” experienced cannot be attributed to the quality of intra- and post-procedural care. Instead, it is only the preoperative care that could be reasonably reflective of weekend practices. As the authors astutely note, patients admitted electively over the weekend for surgery on a weekday may in fact be admitted for preoperative medical optimization. This would suggest that they are indeed at higher risk of a poor outcome after surgery, notwithstanding any optimization.

For elective patients who undergo surgery on the weekend of admission, the increased risk could reflect the overall quality of care on weekends, for all aspects of pre-, intra-, and postoperative care. Alternatively, a weekend surgery in an electively admitted patient may signal that an elective procedure has now become urgent. One further consideration is that elective patients admitted on weekends are slightly more urgent than the average weekday elective patient. Because hospitals and operating rooms have lower occupancy on weekends, shifting an elective case to the weekend may be one way to shorten wait time for a patient whose condition is deteriorating on the wait list. Surgical admissions, although classified in binary manner as “urgent” or “elective,” may exist on the full spectrum of medical acuity.

## The way forward

Previous research has indicated that, in many cases, weekend-admitted patients are sicker than weekday-admitted patients. Large differences in volumes and outcomes for weekend elective patients raise the possibility of confounding on the basis of patient risk profiles. The study by O’Leary and colleagues convincingly demonstrates a large weekend effect for weekend-admitted elective patients who subsequently undergo surgery. However, we need a better understanding of how elective weekend patients may be different. Otherwise, any implementation of widespread staffing or protocol changes would be premature.
